# XPS Depth Profile Analysis of Zn_3_N_2_ Thin Films Grown at Different N_2_/Ar Gas Flow Rates by RF Magnetron Sputtering

**DOI:** 10.1186/s11671-016-1769-y

**Published:** 2017-01-04

**Authors:** M. Baseer Haider

**Affiliations:** 1 Department of Physics, King Fahd University of Petroleum and Minerals, Dhahran, 31261 Saudi Arabia; 2 Affiliate Center of Excellence for Nanotechnology, King Fahd University of Petroleum and Minerals, Dhahran, 31261 Saudi Arabia

**Keywords:** Semiconductors, Magnetron sputtering, Zinc nitride, XPS

## Abstract

Zinc nitride thin films were grown on fused silica substrates at 300 °C by radio frequency magnetron sputtering. Films were grown at different N_2_/Ar flow rate ratios of 0.20, 0.40, 0.60, 0.80, and 1.0. All the samples have grain-like surface morphology with an average surface roughness ranging from 4 to 5 nm and an average grain size ranging from 13 to16 nm. Zn_3_N_2_ samples grown at lower N_2_/Ar ratio are polycrystalline with secondary phases of ZnO present, whereas at higher N_2_/Ar ratio, no ZnO phases were found. Highly aligned films were achieved at N_2_/Ar ratio of 0.60. Hall effect measurements reveal that films are n-type semiconductors, and the highest carrier concentration and Hall mobility was achieved for the films grown at N_2_/Ar ratio of 0.60. X-ray photoelectron study was performed to confirm the formation of Zn–N bonds and to study the presence of different species in the film. Depth profile XPS analyses of the films reveal that there is less nitrogen in the bulk of the film compared to the nitrogen on the surface of the film whereas more oxygen is present in the bulk of the films possibly occupying the nitrogen vacancies.

## Background

Zn_3_N_2_ has recently attracted much attention because of its high transparency, high electron conductivity, and its potential use in optoelectronics, sensors, and renewable energy [1–3]. Zn_3_N_2_ is an n-type semiconductor with either direct or indirect band gap depending upon the deposition techniques and ambient conditions. The optical band gap varies between 1.23 and 3.2 eV which is still controversial [4–7]. Zn_3_N_2_ thin films were first prepared by Kuriyama et al. on quartz substrate by using zinc target and ammonia as the reacting gas [8]. Their Zn_3_N_2_ films were cubic in structure with lattice constant of 0.978 nm and optical band gap of 3.2 eV. They concluded that wide band gap is due to large ionicity of Zn_3_N_2_. Zn_3_N_2_ is a better substitute of Si for fabrication of thin-film transistors (TFT) than other materials like zinc oxide and graphene [9–11]. The fabrication of a reliable p-type ZnO is still an unresolved issue; there have been different attempts of doping ZnO with different group V elements. A relatively stable way of fabricating p-type ZnO could be to replace nitrogen with oxygen in Zn_3_N_2_ [12, 13]. Zinc nitride can be used for deposition of thin transparent, conducting films of p-type ZnO which have excellent applications in light-emitting diodes, laser diodes, and cheap solar cells [9, 13, 14]. These properties make Zn_3_N_2_ an interesting material to study.

In this paper, we report the growth of Zn_3_N_2_ by radio frequency (RF) magnetron sputtering at different nitrogen to argon gas flow rate ratios to find the optimum growth condition and x-ray photoelectron spectroscopy (XPS) depth profile analysis of the grown samples.

## Methods

Thin films of zinc nitride were prepared by the RF magnetron sputtering system on fused silica substrates. The sputtering chamber was first evacuated to an initial pressure of about 1.4 × 10^−5^ mbar. During the growth, the substrate was kept at a temperature of 300 °C and was constantly rotated at an angular speed of 6 RPM to achieve uniform film. Zinc nitride films were deposited by sputtering Zn target (99.9% pure) in the presence of 99.9% pure molecular nitrogen. Samples were grown at different N_2_ gas flow rates ranging from 2 to 10 standard cubic centimeters per minute (SCCM) while the Ar gas flow rate was maintained at 10 SCCM for all the samples. The RF plasma power was kept at 150 W, and sputtering time was about 2 h and 30 min. The growth conditions of different samples are summarized in Table 1.

**Table 1:** Growth condition for zinc nitride thin films grown by RF magnetron sputtering

Sample #	Growth temperature (°C)	Ar flow rate (SCCM)	N_2_ flow rate (SCCM)	N_2_/Ar flow rate ratio
1	300	9	2	0.22
2	300	10	4	0.40
3	300	10	6	0.60
4	300	10	8	0.80
5	300	10	10	1.0

The crystallinity of the film was analyzed by x-ray diffraction by Shimadzu XRD-6000 diffractometer using Cu Kα radiations of wavelength 1.548 Å. Surface morphology of the films was studied by the atomic force microscope (AFM) using non-contact or tapping mode of Veeco Innova diSPM. Gwyddion software was used to analyze the AFM images and estimate the grain size from the AFM images. Three-point leveling was applied to remove the unevenness in the appearance of the raw image that is due to small tilt in the substrate. Laplacian algorithm was used to mark the grains and estimated the grain size by selecting the appropriate threshold that would result in a maximum number of grains in the grain statistics. Average threshold value for our samples was around 40%. Electrical properties of the grown films were studied by Hall effect measurements by four probe Van der Pauw method at room temperature using ECOPIA HMS3000 Hall effect system. Silver paint was used to make the contacts for Hall effect measurements. After the growth, samples were transported in the air to the XPS chamber. After the samples were placed in the XPS chamber, the chamber was pumped down to a pressure of about 5 × 10^-10^ mbar. XPS is a surface sensitive technique and can probe first few layers of the sample surface and is used for the elemental analysis and bonding nature of the constituent species in the sample. Thermo Fisher ESCALAB 250Xi spectrometer was used for XPS analysis which has Al-K_α_ as an x-ray source. Depth profile XPS study was performed by etching the surface by Ar ion beam for about 30 s repeatedly, and XPS spectra was taken after every etching cycle.

## Results and Discussion

**Fig. 1:**
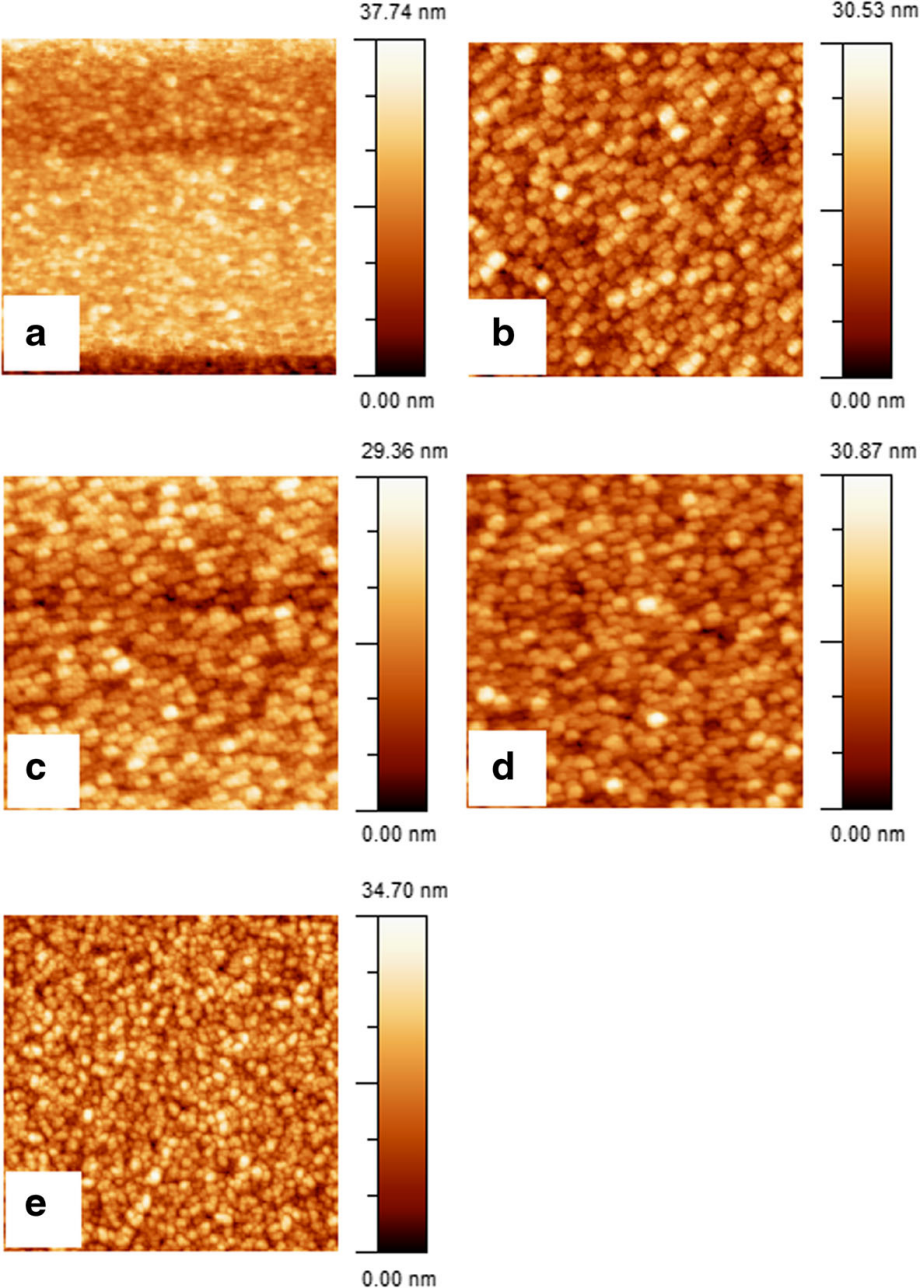
AFM images (2 × 2 μm^2^ size) of samples grown at N_2_/Ar gas flow rate of **a** 0.22, **b** 0.40, **c** 0.60, **d** 0.80, and **e** 1.0

Surface morphology of the grown zinc nitride thin films was obtained by atomic force microscopy. Shown in Fig. 1 are 2 × 2 μm^2^-size AFM images of the samples grown at N_2_/Ar flow rate ratio of 0.22, 0.40, 0.60, 0.80, and 1.0. Surface of the sample grown at N_2_/Ar ratio of 0.22 has 3D grain-like morphology, and the root mean square roughness was found to be 4.7 nm with an average grain size of about 14 nm. The root mean square roughness of the sample grown at N_2_/Ar ratio of 0.40 is 4.4 nm with an average grain size of 13 nm. Here, we note that increase in the N_2_/Ar ratio during the growth has a little effect on the surface roughness and the gain size of the two samples. The surface root mean square roughness of the sample grown at N_2_/Ar ratio of 0.60 is 4.3 nm, and the average grain size is about 14 nm. AFM image of the sample grown at N_2_/Ar ratio of 0.80 reveals the root mean square roughness of 4.2 nm with an average grain size of about 16 nm. The root mean square roughness of the sample grown at N_2_/Ar ratio of 1.0 is about 4.9 nm, and the average grain size is about 14 nm. A summary of the average grain size of different samples as measured by AFM images is provided in Table 2.

**Table 2:** Average grain size and Hall effect data of all the samples

Sample #	N_2_/Ar flow rate ratio	Average grain size (nm)	Carrier concentration (cm^−3^)	Hall mobility (cm^2^/V s)
1	0.22	14	−1.38 × 10^18^	0.1125
2	0.40	13	−1.53 × 10^20^	0.01034
3	0.60	14	−1.01 × 10^21^	0.3012
4	0.80	16	−2.601 × 10^20^	0.1028
5	1.0	13	−2.02 × 10^21^	0.0084

The AFM study of the grown films reveals that all the samples have grain-like surface morphology with an average surface roughness of about 4–5 nm. A little difference in the surface roughness or grain size was observed between the samples grown at different N_2_/Ar gas flow rate ratios.

**Fig. 2:**
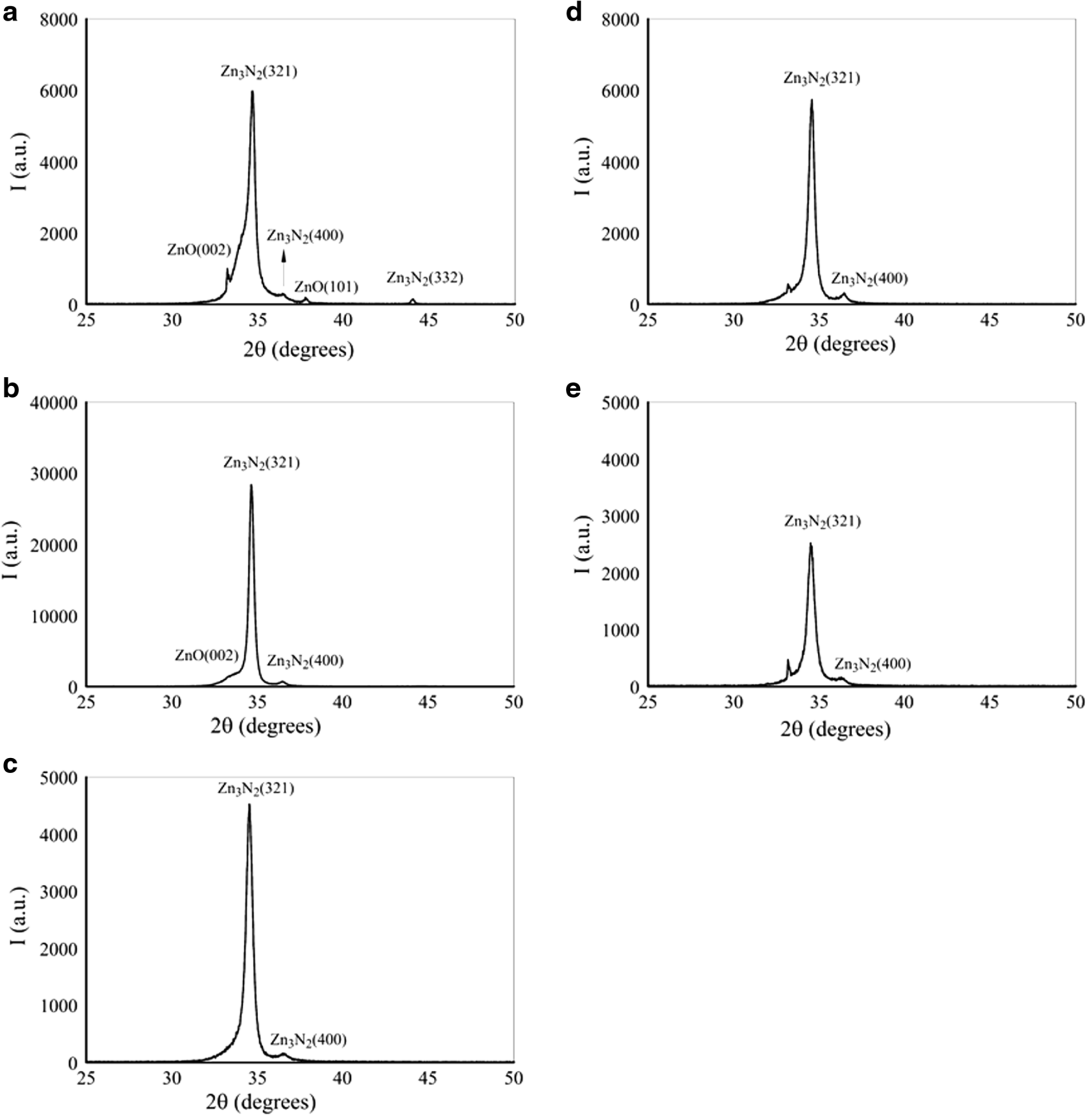
X-ray diffraction patterns of samples grown at N_2_/Ar gas flow rate ratio of **a** 0.22, **b** 0.40, **c** 0.60, **d** 0.80, and **e** 1.0

X-ray diffraction was performed to measure the crystallinity of the samples. Shown in Fig. 2a is the XRD pattern of the sample grown at N_2_/Ar flow rate ratio of 0.22. The diffractogram shows a main peak around 2*θ* = 34.68° corresponding to Zn_3_N_2_ (321) and a few small peaks around 34.2°, 36.5°, 37.9°, and 44.1° corresponding to ZnO (002), Zn_3_N_2_ (400), ZnO (101), and Zn_3_N_2_ (332), respectively. This indicates that the sample grown at N_2_/Ar ratio of 0.22 is a polycrystalline sample, and some ZnO phases are also present. It has been reported that Zn_3_N_2_ can be hydrolyzed rather easily because the binding energy of Zn–O is higher than Zn–N, so Zn–O bond is preferable compared to the Zn–N bond [15]. Shown in Fig. 2b is the XRD pattern of sample grown at N_2_/Ar ratio of 0.40. The XRD pattern shows a main peak at 34.6° corresponding to Zn_3_N_2_ (321) and a small peak around 36.4° corresponding to Zn_3_N_2_ (004). A shoulder on the left side of the main Zn_3_N_2_ (321) is present in this XRD pattern as well that corresponds to ZnO (002) peak. Whereas the other phases of ZnO and Zn_3_N_2_, that were present in the XRD pattern of the sample grown at N_2_/Ar ratio of 0.22, are absent in the sample grown at N_2_/Ar ratio of 0.40. This indicates that by increasing the N_2_/Ar ratio, crystallinity of the sample has improved as well as ZnO phases are reduced in number and intensity. Shown in Fig. 2c is the XRD pattern of the sample grown at N_2_/Ar ratio of 0.60. The XRD pattern shows a main peak at 34.6° corresponding to Zn_3_N_2_ (321) and a small peak around 36.52° corresponding to Zn_3_N_2_ (004). It can be seen from the XRD pattern that the shoulder of the Zn_3_N_2_ (321) peak has been completely disappeared. This indicates that the sample is completely Zn_3_N_2_ and does not contain any secondary phases that were present in the samples grown at lower N_2_/Ar ratios. The XRD pattern of the sample grown at N_2_/Ar ratio of 0.80 and 1.0 are shown in Fig. 2d, e, respectively. Both of these patterns contain main Zn_3_N_2_ (321) peak around 34.6°, whereas a small peak of Zn_3_N_2_ (400) peak is also present in both the samples. Additionally, both of these samples also contain an unknown sharp peak around 33.2°, and this peak grows with the increase in N_2_/Ar ratio.

The XRD patterns of the grown samples reveal that all the samples contain main peak of Zn_3_N_2_ (321). We observed that the appearance of different phases of Zn_3_N_2_ in the film depend upon the N_2_/Ar ratio. The sample grown at the lowest N_2_/Ar ratio (0.22) has the highest number of Zn_3_N_2_ phases along with some ZnO phases, whereas sample grown at N_2_/Ar ratio of 0.60 contains only Zn_3_N_2_(321) along with a satellite Zn_3_N_2_(004) peak with no ZnO phases. Whereas the XRD patterns of the samples grown at even higher N_2_/Ar ratio contain another unknown peak at 33.2°. This indicates that the best growth condition for Zn_3_N_2_ films is achieved when grown at N_2_/Ar gas ratio of 0.60.

The electrical and Hall effect measurements reveal that all Zn_3_N_2_ samples grown at different N_2_/Ar ratios have n-type conductivity. The carrier concentration of the films increases with the increase in N_2_ flow rate during the deposition from ~10^18^ to 10^21^ cm^−3^. A summary of the carrier concentration and Hall mobility of the grown samples is shown in Table 2. The highest Hall mobility and carrier concentration were found for the sample grown at N_2_/Ar ratio of 0.60. By further increasing the N_2_/Ar ratio, the carrier concentration slightly reduces but the Hall mobility is reduced 300%. This indicates that N_2_/Ar ratio of 0.60 results in the optimum growth condition with the highest carrier concentration and Hall mobility. Higher N_2_/Ar gas flow rate ratio results in higher chamber pressure during the growth and that would reduce the mean free path of the sputtering species which in this case is zinc. Low density of Zn atoms on the substrate would result in Zn vacancies and interstitials, and this could be the reason for low carrier concentration and low Hall mobility.

To investigate the presence of different species in the film, we have performed an XPS study of the films. A wide scan XPS survey was performed to confirm the presence of different species in the film then detailed high-resolution spectra were performed for Zn2p, N1s, and O1s level regions. We have also performed depth-profiling study of our zinc nitride films to investigate the presence of different species at different levels in the film. The film was etched by low-energy Ar ions for 30 s repeatedly until the films substrate interface was reached. After each etching, high-resolution XPS spectra of Zn2p, N1s, and O1s level regions were obtained.

**Fig. 3:**
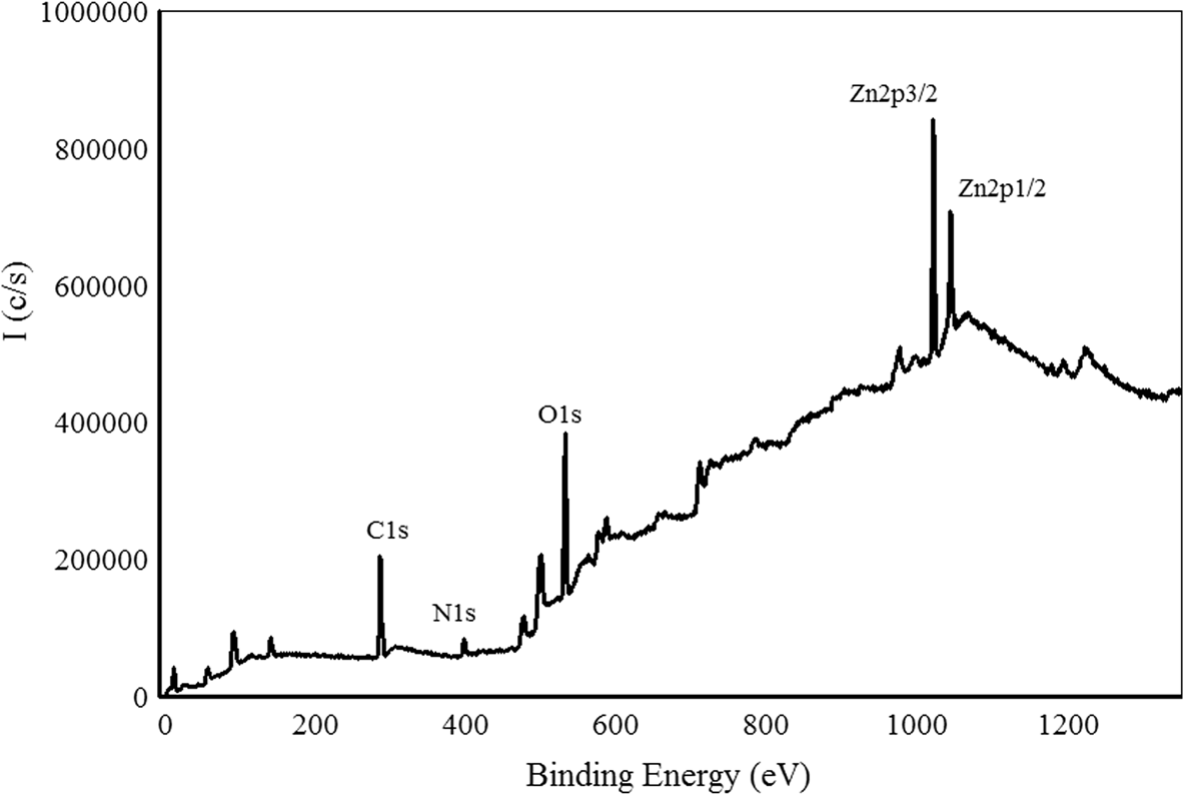
XPS full scan survey of sample grown at N_2_/Ar gas flow rate ratio of 0.60 before etching

**Fig. 4:**
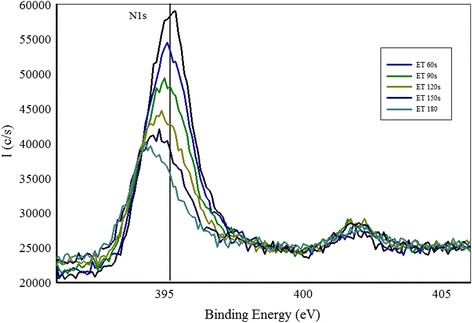
High resolution XPS scan of a region containing N1s peak taken at different etching levels

Shown in Fig. 3 is a wide scan survey of the film grown at N_2_/Ar ratio of 0.60. The spectrum reveals the presence of Zn2p, O1s, N1s, and C1s levels regions. Carbon peak appears due to hydrocarbon contaminants deposited after the growth when sample was in the air before being transferred to the XPS chamber. C1s peak position at 284.8 eV is used as a binding energy reference. Due to spin-orbit coupling, Zn2p level is split into two peaks Zn2p3/2 and Zn2p1/2. Shown in Fig. 4 is the high-resolution XPS spectrum of Zn2p3/2 level region obtained after 30 s of etching. Zn peak has been deconvoluted into two peaks that are positioned at 1021.1 and 1020.4 eV that can be attributed to Zn–O and Zn–N bonds, respectively [16].

**Fig. 5:**
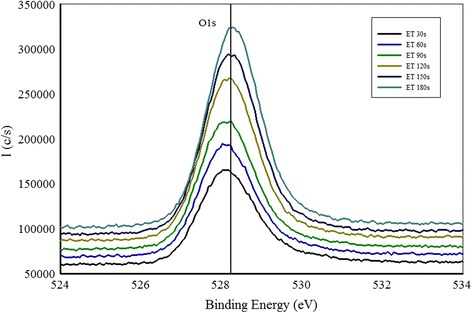
High resolution XPS scan of a region containing O1s peak taken at different etching levels

**Fig. 6:**
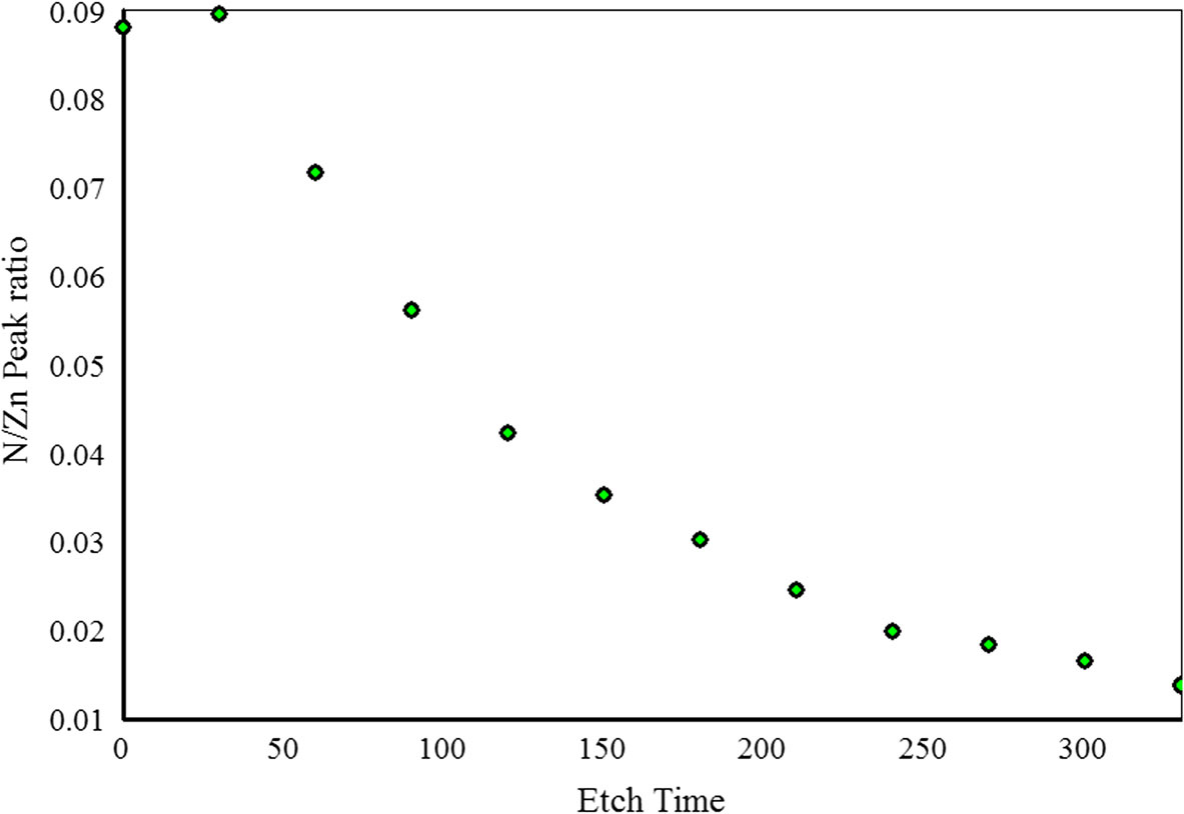
N1s to Zn2p3/2 XPS peak intensities ratio of sample with N_2_/Ar ratio of 0.60 at different etching levels

**Fig. 7:**
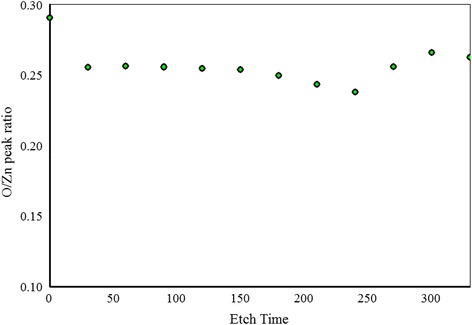
O1s to Zn2p3/2 XPS peak intensities ratio of sample with N_2_/Ar ratio of 0.60 at different etching levels

Shown in Fig. 4 are the N1s spectra of the sample grown at N_2_/Ar ratio of 0.60 taken at different etching levels. We observe that N1s peak is positioned at 395.8 eV that shows a large chemical shift compared to free amine N_2_ peak at 398.8 eV. This indicates that Zn–N bonds are formed. Another small peak is observed around 402 eV that can be due to N–N bond formation in the film. Another interesting phenomenon is observed that the intensity of N1s peak is decreasing after every etching level and finally the peak disappears when the film substrate interface is reached. This indicates that the amount of N in the film is decreasing in the bulk of the film. A slight shift of the N1s peak toward the lower binding energy is observed for the spectra taken deeper in the film. This indicates the change in chemical environment of N deeper in the film compared to the chemical environment of N at or near the surface. An increase in the intensity of the O1s peak is observed at different etching levels as shown in Fig. 5, and no shift in O1s peak position is observed for the spectra taken deeper in the film. A plot of N1s/Zn2p3/2 peak intensity ratio as a function of etching time is drawn in Fig. 6. We notice that there is a continuous decrease in the N1s/Zn2p3/2 peak intensity ratio at different etching levels whereas O1s/Zn2p3/2 peak intensity ratio is almost constant throughout the film as shown in Fig. 7. Similar trend of reduced N1s peak intensity and increased O1s peak intensity was observed for all the other samples.

Such a decrease in the intensity of nitrogen XPS peak as a function of depth has not been reported before in the literature. A possible explanation could be that the closer to the substrate-film interface, there are more nitrogen vacancies due to stress in the film arising from the lattice mismatch between the substrate and the film. As the film grows, the stress in the film is reduced and as a result, there is a reduction in the nitrogen vacancies. A rigorous theoretical work is required to understand the behavior of nitrogen in zinc nitride.

## Conclusions

Zn_3_N_2_ thin films were prepared by RF magnetron sputtering of Zn target in nitrogen-argon mixture of gases at substrate temperature of 300 °C. It was observed that the structural and electronic properties are extremely dependent on N_2_/Ar gas flow rate ratio during the growth. The samples grown at lower N_2_/Ar ratio (less than 0.60) seem to be polycrystalline and contain Zn_3_N_2_ and ZnO phases. Whereas the sample grown at N_2_/Ar ratio of 0.60 is highly crystalline compared to the other samples grown at lower or higher N_2_/Ar ratio. The grain like surface morphology was observed for all the samples grown at different N_2_/Ar ratios with the average surface roughness ranging from 4 to 5 nm and average grain size ranging from 13 to 16 nm. Electrical measurements show that all the films grown at different N_2_/Ar ratios are n-type and carrier concentration as well as Hall mobility increase with the increase in the N_2_/Ar ratio until this ratio reaches 0.60. Further raise in the N_2_/Ar ratio results in film with lower carrier concentration and Hall mobility. This indicates that optimum growth condition for Zn_3_N_2_ sample grown at 300 °C by RF magnetron sputtering is achieved at N_2_/Ar gas flow rate ratio of 0.60. XPS study of the films confirms the formation of Zn–N bonds, and it was found that the intensity of N1s peak reduces whereas O1s peak intensity increases as we go deeper in the film. This indicates more nitrogen vacancies are formed in the beginning of the film, and the atmospheric oxygen compensates those vacancies. Nitrogen vacancies deeper in the film can be attributed to the greater stress in the film closer to the substrate due to film-substrate lattice mismatch.

## Abbreviations

XPS: X-ray photoelectron spectroscopy
Zn_3_N_2_: Zinc nitride
RF: Radio frequency
ZnO: Zinc oxide
RPM: Revolutions per minute
SCCM: Standard cubic centimeters per minute
AFM: Atomic force microscope
XRD: X-ray powder diffraction

## References

[1] Jiangyan W, Jinliang Y, Wei Y, Tang L (2012). Structural and optical properties of Zn_3_N_2_ films prepared by magnetron sputtering in NH3–Ar mixture gases. J Semicond.

[2] Bhattacharyya SR, Ayouchi R, Pinnisch M, Schwarz R (2012). Transfer characteristic of zinc nitride based thin film transistors. Phys Status Solidi.

[3] Jiang N, Georgiev DG, Jayatissa AH (2013). The effects of the pressure and the oxygen content of the sputtering gas on the structure and the properties of zinc oxy-nitride thin films deposited by reactive sputtering of zinc. J Semicond Sci Technol.

[4] Long R, Dai Y, Yu L, Guo M, Huang B (2007). Structural, electronic, and optical properties of oxygen defects in Zn_3_N_2_. J Phys Chem.

[5] Xing GZ, Wang DD, Yao B, Ah Qune LFN, Yang T (2010). Structural and electrical characteristics of high quality (100) orientated-Zn_3_N_2_ thin films grown by radio-frequency magnetron sputtering. J Appl Phys.

[6] Zong F, Ma H, Ma J, Du W, Zhang X, Xiao H, Ji F (2005). Structural properties and photoluminescence of zinc nitride nanowires. Appl Phys Lett.

[7] Du W, Zong F, Ma H, Zhang M, Feng X, Li H, Zhang Z, Zhao P (2006). Optical band gap of zinc nitride films prepared by reactive rf magnetron sputtering. Cryst Res Technol.

[8] Kuriyama K, Takahashi Y, Sunohara F (1993). Optical band gap of Zn_3_N_2_ films. Phy Rev B.

[9] Georgiev DG, Moening JP, Naleshwar S, Ahalapitiya AH. Microstructure and electronic properties of zinc nitride thin films. IEEE Nanotechnology Materials and Devices Conference 2009. 978-1-4244-4696-4/09.

[10] Garcia Nunez C, Pau JL, Hernandez MJ, Cervera M, Ruiz E, Piqueras J (2011). On the true optical properties of zinc nitride. Appl Phys Lett.

[11] Aperathitis E, Kambilafka V, Modreanu M (2009). Properties of n-type ZnN thin films as channel for transparent thin film transistors. Thin Solid Films.

[12] Nunez CG, Pau JL, Hernandez MJ, Cervera M, Ruiz E, Piqueras J (2012). On the zinc nitride properties and the unintentional incorporation of oxygen. Thin Solid Films.

[13] Fu- Jian Z, Lei MH, Wei L, Wei D, Xi- Jian Z, Hong-Di X, Jin M, Feng J, Cheng-shan X, Zhao ZH (2005). Thermal decomposition behaviour of Zn_3_N_2_ powder. Chin Phys Lett.

[14] Voulgaropoulou P, Dounis S, Kambilafka V, Androulidaki M, Ruzinsky M, Saly V, Prokien P, Viskadourakis Z, Tsagaraki K, Aperathitis E (2008). Optical properties of zinc nitride thin films fabricated by rf-sputtering from ZnN target. Thin Solid Films.

[15] Futsuhara M, Yoshioka K, Takai O (1998). Structural, electrical and optical properties of zinc nitride thin films prepared by reactive rf magnetron sputtering. Thin Solid Films.

[16] Gangil S, Nakamura A, Yamamoto K, Ohashi T, Temmyo J (2008) Fabrication and EL Emission of ZnO-Based Heterojunction Light-Emitting Devices. J Korean Phys Soc 53:212–217

